# Supplementary material for: Spatiotemporal forecasting of vertical track alignment with exogenous factors
Source: Sci Rep. 2023 Feb 9;13:2354. doi: 10.1038/s41598-023-29303-7 (PMC9911736; doi:10.1038/s41598-023-29303-7)
Supplement: Supplementary file 1 — Supplementary Information. [file 41598_2023_29303_MOESM1_ESM.pdf]

Supplementary material

S.1 Maintenance operations for vertical alignment correction

We list the nine maintenance operations used for forecasting vertical track alignment in Table 4 of the main manuscript. These nine operations are categorized by merging more detailed operations. Here we show the full list of the maintenance operations including the detailed operations in Table S1. For example, the category of sleeper maintenance includes sleeper replacement, loose sleeper repair, and so on.

Detailed maintenance	Merged category
Uneven fixing	Uneven fixing
Tamping by multiple tie tamper	Tamping by multiple tie tamper
Manual tamping	Manual tamping
Ballast replacement	Ballast replacement
Right rail replacement	Right rail replacement
Left rail replacement	Left rail replacement
Sleeper replacement<br>Sleeper installation<br>Loose sleeper repair<br>Sleeper alignment<br>Sleeper removal<br>Sleeper relocation	Sleeper maintenance
Remediation of mud-pumping	Remediation of mud-pumping
Expansion joint replacement<br>Turnout replacement<br>Glued insulation joint replacement<br>Rail grinding	Others

Supplementary Table S1: Full list of maintenance operations for vertical alignment correction

S.2 Loss curves for training and validation data

Figure S1 show the loss curves of ConvLSTM, GRU, and LSTM in the comparison experiment. The blue and orange lines show the losses for training and validation data, respectively. As shown in Figure S1, the loss curves for both the training and validation data decrease as the epoch progresses. These results indicate overfitting does not occur for all ConvLSTM, GRU, and LSTM.

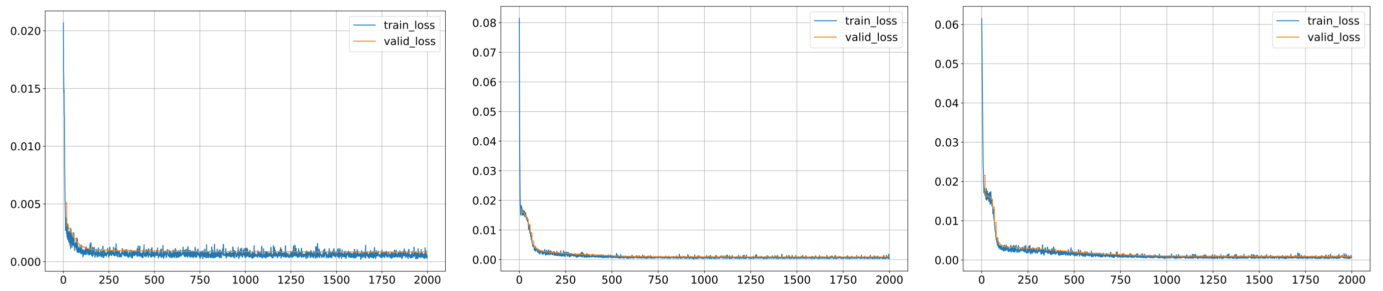

(a) ConvLSTM (b) GRU (c) LSTM

Supplementary Figure S1: The loss curves of (a) ConvLSTM, (b) GRU, and (c) LSTM.

S.3 Ablation study on exogenous factors for LSTM and GRU

Tables S2, S3 and S4 show the results of the ablation study on the exogenous factor for LSTM and GRU. The results are similar to those of ConvLSTM (see Tables 9, 10, and 11 in the main manuscript). For example, comparing "with-all" and "without-maintenance", "without-maintenance" shows a higher RMSE and a lower accuracy for both the entire dataset and the dataset with thresholds $\alpha = -4, -6$ [mm]. Therefore, the maintenance records are also significant for forecasting in LSTM and GRU. Additionally, the results prove that ConvLSTM outperforms LSTM and GRU (see Tables 9, 10, and 11 in the main manuscript).

Case name	Exogenous data						RMSE(mm) (↓)		
	Maintenance	Structure	Rail joint	Ballast age	Tonnage	Rainfall	Entire	< -4mm	< -6mm
w/ all	✓	✓	✓	✓	✓	✓	0.302	1.091	2.477
w/o maintenance		✓	✓	✓	✓	✓	0.369	1.226	2.651
w/o structure	✓		✓	✓	✓	✓	0.305	1.114	2.526
w/o rail joint	✓	✓		✓	✓	✓	0.302	1.104	2.469
w/o ballast age	✓	✓	✓		✓	✓	0.305	1.091	0.247
w/o tonnage	✓	✓	✓	✓		✓	0.305	1.090	2.417
w/o rainfall	✓	✓	✓	✓	✓		0.300	1.079	2.402
w/o all							0.369	1.194	2.582

(a) LSTM

Case name	Exogenous data						RMSE(mm) (↓)		
	Maintenance	Structure	Rail joint	Ballast age	Tonnage	Rainfall	Entire	< -4mm	< -6mm
w/ all	✓	✓	✓	✓	✓	✓	0.300	1.085	2.406
w/o maintenance		✓	✓	✓	✓	✓	0.366	1.270	2.762
w/o structure	✓		✓	✓	✓	✓	0.299	1.091	2.416
w/o rail joint	✓	✓		✓	✓	✓	0.299	1.083	2.398
w/o ballast age	✓	✓	✓		✓	✓	0.299	1.093	2.414
w/o tonnage	✓	✓	✓	✓		✓	0.299	1,089	2.402
w/o rainfall	✓	✓	✓	✓	✓		0.299	1.093	2.437
w/o all							0.365	1.285	2.828

(b) GRU

Supplementary Table S2: Results of the ablation study (RMSE) for (a) LSTM and (b) GRU. The RMSE is calculated with both the entire data and data with the threshold levels $\alpha = -4, -6$[mm].

Case name	Exogenous data						Accuracy(%) (↑)		
							< -4mm		
	Maintenance	Structure	Rail joint	Ballast age	Tonnage	Rainfall	±0.3mm	±0.5mm	±1.0mm
w/ all	✓	✓	✓	✓	✓	✓	56.55	72.51	85.13
w/o maintenance		✓	✓	✓	✓	✓	05.14	22.74	70.60
w/o structure	✓		✓	✓	✓	✓	57.26	71.65	84.07
w/o rail joint	✓	✓		✓	✓	✓	57.05	72.38	84.70
w/o ballast age	✓	✓	✓		✓	✓	59.51	73.16	85.03
w/o tonnage	✓	✓	✓	✓		✓	59.98	73.48	85.12
w/o rainfall	✓	✓	✓	✓	✓		63.66	75.83	85.83
w/o all							05.52	25.08	73.19

(a) LSTM

Case name	Exogenous data						Accuracy(%) (↑)		
							< -4mm		
	Maintenance	Structure	Rail joint	Ballast age	Tonnage	Rainfall	±0.3mm	±0.5mm	±1.0mm
w/ all	✓	✓	✓	✓	✓	✓	61.21	74.96	86.01
w/o maintenance		✓	✓	✓	✓	✓	07.56	23.32	64.36
w/o structure	✓		✓	✓	✓	✓	63.55	76.22	86.01
w/o rail joint	✓	✓		✓	✓	✓	63.40	76.11	85.97
w/o ballast age	✓	✓	✓		✓	✓	63.66	75.83	85.71
w/o tonnage	✓	✓	✓	✓		✓	63.83	76.08	85.76
w/o rainfall	✓	✓	✓	✓	✓		64.48	76.59	86.26
w/o all							09.24	24.70	64.02

(b) GRU

Supplementary Table S3: Results of the ablation study (accuracy) for (a) LSTM and (b) GRU. The accuracy is calculated with tolerance $\varepsilon = 0.3, 0.5, 1.0$[mm] on the data with the evaluation threshold levels $\alpha = -4$[mm].

Case name	Exogenous data						Accuracy(%) (↑)		
	Maintenance	Structure	Rail joint	Ballast age	Tonnage	Rainfall	< -6mm		
							±0.3mm	±0.5mm	±1.0mm
w/ all	✓	✓	✓	✓	✓	✓	05.44	17.67	46.02
w/o maintenance		✓	✓	✓	✓	✓	00.00	00.00	01.36
w/o structure	✓		✓	✓	✓	✓	06.21	21.75	42.52
w/o rail joint	✓	✓		✓	✓	✓	22.14	35.92	52.82
w/o ballast age	✓	✓	✓		✓	✓	04.66	20.58	45.05
w/o tonnage	✓	✓	✓	✓		✓	09.71	24.66	46.02
w/o rainfall	✓	✓	✓	✓	✓		15.15	30.10	48.74
w/o all							00.00	00.00	03.30

(a) LSTM

Case name	Exogenous data						Accuracy(%) (↑)		
	Maintenance	Structure	Rail joint	Ballast age	Tonnage	Rainfall	< -6mm		
							±0.3mm	±0.5mm	±1.0mm
w/ all	✓	✓	✓	✓	✓	✓	16.12	30.87	49.71
w/o maintenance		✓	✓	✓	✓	✓	00.00	00.00	01.55
w/o structure	✓		✓	✓	✓	✓	24.08	36.50	51.84
w/o rail joint	✓	✓		✓	✓	✓	23.88	35.34	51.65
w/o ballast age	✓	✓	✓		✓	✓	21.36	33.20	50.29
w/o tonnage	✓	✓	✓	✓		✓	22.33	34.17	51.07
w/o rainfall	✓	✓	✓	✓	✓		20.97	34.17	51.46
w/o all							00.00	00.00	01.55

(b) GRU

Supplementary Table S4: Results of the ablation study (accuracy) for (a) LSTM and (b) GRU. The accuracy is calculated with tolerance $\varepsilon = 0.3, 0.5, 1.0$[mm] on the data with the evaluation threshold levels $\alpha = -6$[mm].

S.4 Layer tuning of LSTM and GRU

To determine the best architectures for LSTM and GRU, we examine the forecasting performance by changing the number of layers for LSTM and GRU. Tables S5 and S6 show the RMSE and accuracy of the tuning results, respectively. In the tables, the number of layers means that the architecture consists of that number of layers. We also show the results by ConvLSTM for comparison. In each case, ConvLSTM provides better RMSE and accuracy than those obtained by tuning LSTM and GRU.

Num. layers	RMSE(mm) (↓)		
	Entire	< -4mm	< -6mm
1	0.300	1.096	2.451
2	0.302	1.091	2.411
3	0.304	1.107	2.460
4	0.315	1.137	2.538
ConvLSTM	0.293	1.071	2.343

(a) LSTM

Num. layers	RMSE(mm) (↓)		
	entire	< -4mm	< -6mm
1	0.300	1.097	2.428
2	0.300	1.085	2.406
3	0.299	1.090	2.412
4	0.299	1.083	2.399
ConvLSTM	0.293	1.071	2.343

(b) GRU

Supplementary Table S5: RMSE results by tuning the number of layers for (a) LSTM and (b) GRU.

Num. layers	Accuracy(%) (↑)					
	< -4mm			< -6mm		
	±0.3mm	±0.5 mm	±1.0 mm	±0.3 mm	±0.5 mm	±1.0 mm
1	62.02	75.12	85.58	10.87	26.99	48.54
2	56.55	72.52	85.14	05.44	17.67	46.21
3	56.03	71.28	84.15	03.11	19.81	43.69
4	47.04	64.86	81.84	00.78	05.83	34.56
ConvLSTM	66.48	77.82	87.35	26.02	37.28	54.76

(a) LSTM

Num. layers	Accuracy(%) (↑)					
	< -4mm			< -6mm		
	±0.3mm	±0.5 mm	±1.0 mm	±0.3 mm	±0.5 mm	±1.0 mm
1	60.74	74.27	85.04	18.06	29.51	47.18
2	61.21	74.96	86.01	16.12	30.87	49.71
3	62.53	75.65	85.71	23.30	34.56	51.07
4	62.07	75.18	85.72	18.83	31.07	48.54
ConvLSTM	66.48	77.82	87.35	26.02	37.28	54.76

(b) GRU

Supplementary Table S6: Accuracy results by tuning the number of layers for (a) LSTM and (b) GRU.
